# Second Primary Renal Cell Carcinoma With Nonrenal Malignancies: An Analysis of 118 Cases and a Review of Literature

**DOI:** 10.3389/fonc.2021.780130

**Published:** 2021-11-25

**Authors:** Jinchao Chen, Nienie Qi, Hua Wang, Zongping Wang, Yedie He, Shaoxing Zhu

**Affiliations:** ^1^ Department of Urologic Surgery, The Cancer Hospital of the University of Chinese Academy of Sciences, Zhejiang Cancer Hospital, Institute of Basic Medicine and Cancer (IBMC), Chinese Academy of Sciences, Hangzhou, China; ^2^ Department of Urology, The Affiliated Hospital of Xuzhou Medical University, Xuzhou, China

**Keywords:** renal cell carcinoma, multiple primary malignancy, diagnosis, treatment, renal metastases

## Abstract

**Objectives:**

To evaluate the nature, diagnosis, treatment and prognosis of second primary renal cell carcinoma (SPRCC).

**Materials and Methods:**

We retrospectively collected data from 118 patients with SPRCC. Clinical characteristics, imaging features and treatments were analyzed and comparisons between SPRCC and renal metastases (RM) were made.

**Results:**

SPRCC accounts for 11.4% of all RCC. The most common types of extrarenal malignancies included lung, colorectal, breast and gynecological cancers. The median age was 58.5 years old, and 61.0% (72/118) of the patients were male. About 5.1% of the patients presented with symptoms. The average tumor diameter was 4.4 cm (1-8.4 cm). The diagnostic specificity of enhanced computed tomography (CT) was 80.1%. When comparing with RM, more patients with stage I–II extrarenal malignancy and less patients with bilateral, multiple, and endogenic renal masses on computed tomography were found in the SPRCC group. A total of 110 SPRCC patients underwent surgery, including 48 radical nephrectomies and 62 partial nephrectomies. The median overall survival time was 117 months. Female, asymptomatic status, no distant metastasis, and surgical treatment predicted a better survival.

**Conclusions:**

SPRCC are not uncommon, and it should be considered during the follow-up of patients with nonrenal malignancy. The differential diagnosis between SPRCC and RM was mainly based on imaging and puncture biopsy.

## Introduction

The second primary cancer (SPC) refers to the malignancy found at the same time with the primary cancer or later, which should exclude metastasis and recurrence (1). SPC is one of the major prognostic factors among cancer survivors, and it is estimated to be the sixth most common form of malignancy worldwide (2). The development of diagnostic and treatment technologies for cancer has resulted in a growing population of cancer survivors, and the incidence and mortality from SPC are likely to increase (3). In the United States, the proportion of cancer survivors among the total population is estimated to be 3.5%, and approximately 10% of newly diagnosed cancers develop in cancer survivors (4).

Second Primary Renal Cell Carcinoma (SPRCC) is a common type of SPC (5). Types of primary malignancies associated with RCC include bladder, prostate, colorectal, lung cancers, cutaneous malignant melanoma (MM) and non-Hodgkin’s lymphoma (NHL) (5). Its occurrence may reflect potential immune deficiencies, gene mutations, exposure to carcinogens, the sequelae of cancer and its treatment or improved diagnostics (6). In the past, most reports on SPRCC consisted of either single-institution studies or purely register-based data (5, 7–10). However, the nature of these special RCC, as well as their diagnosis, treatment and prognosis has not been described in detail. Furthermore, when faced with a renal mass in patients with nonrenal malignancy, the differential diagnosis of SPRCC from renal metastases was critical.

Here, we analyzed the data of 118 patients with SPRCC, and intended to describe their clinical features, diagnosis and treatment methods. Furthermore, we also made a comparison between RM and SPRCC.

## Materials and Methods

Studies involving human participants were reviewed and approved by the Medical Ethics Committee of Zhejiang Cancer Hospital. The database records of patients diagnosed with RCC at Zhejiang Cancer Hospital from 1 January 2009 to 31 December 2019 were reviewed. Nonrenal primary malignancies were recorded and classified as antecedent and synchronous, and the patients who were diagnosed after RCC (subsequent) were excluded. Synchronous malignancies were defined as those diagnosed concurrently or within 6 months of diagnosis.

We retrospectively collected 118 cases of SPRCC. The clinical data of these patients were collected, including demographic data, pathology and treatment of nonrenal malignancies, symptoms, imaging features (enhanced computed tomography, CT), and pathology and treatment of RCC. The number, location, size, and enhancement characteristics of the renal lesions were evaluated by a specialized radiologist. An exophytic mass was one that protruded by > 50% outside the kidney. The included cases (n = 106) were followed up to record the recurrence, metastasis, and survival status. The overall survival (OS) of the patients refers to the time from the diagnosis of RCC to the last follow-up or death. Thirty-five patients with renal metastases (RM) were also studied, and the methods were described in our previous study (11).

Classified variables and continuous variables were tested by chi-square test and t-test, respectively. OS was calculated using the Kaplan-Meier method for the study population. Multivariate Cox regression analysis was used to explore factors influencing prognosis. All statistical analyses were performed using the SPSS V.19 (IBM) statistical software. Statistical significance was set at p < 0.05.

## Results

### Patients’ Characteristics

The demographic details of the SPRCC are presented in **Table 1**. SPRCC accounts for 11.4% of all RCC. The median age was 58.5 years old, and 61.0% (72/118) of the patients were male. Around 44.9% and 32.2% of the patients had smoking history and family history of cancers, respectively. The mean body mass index (BMI) of SPRCC patients was 23.9 kg/m^2^. And, about 5.1% of the patients presented with symptoms.

**Table 1:** Demographics and clinical characteristics of patients with second primary renal cell carcinoma and with those of renal metastases.

		Second primary renal cell carcinoma	Renal metastases	P value
Number		118	35	
Sex	Male	72	23	0.62
	Female	46	12	
Age		58.5	62.0	0.084
Smoking history	Yes	53	15	0.93
	No	65	19	
Family history of malignancy	Yes	38	9	0.47
	No	80	26	
Body Mass Index (kg/m^2^)		23.9	21.9	0.002
Symptom	Yes	6	14	<0.001
	No	112	21	
Duration between twice cancer onsets (month)		33.5	29.4	0.81
Synchronous		73	6	<0.001
Metachronous		45	29	
Diameter of renal mass (mean, cm)		3.7	4.4	0.073
Renal biopsy	Yes	7	16	<0.001
	No	111	18
Treatment	Renal surgery	110	21	<0.001
	No renal surgery	8	14
Stage of extrarenal malignancies	I/II	71	16	0.012
	III/IV	23	15
	Missing	24	4
History ofchemotherapy or radiotherapy	Yes	53	25	0.01
	No	65	11

The origin sites of primary nonrenal malignancies of patients with SPRCC consisted of lung (19.5%), colorectal (14.4%), breast (10.2%), gynecological cancers (9.3%), stomach (8.5%), thyroid (8.5%) and nasopharynx (8.5%). Synchronous SPRCC was found in 73 patients (61.9%), and the mean duration between twice cancer onsets was 33.5 months.

### Features of SPRCC

Of these patients with SPRCC, 113 (95.8%) had localized RCC and five (4.2%) had metastatic RCC. All of the cases were unilateral and 96.6% were solitary. The average tumor size was 4.4 cm (1-8.4 cm) (**Table 1**). 116 patients underwent enhanced CT scan, and about 80.1% were diagnosed with SPRCC, however, up to 12.9% were diagnosed with renal metastasis (**Table 2**).

**Table 2:** Radiological characteristics of patients with second primary renal cell carcinoma and with those of renal metastases.

Radiological evaluation		Second primary renal cell carcinoma	Renal metastases	P value
Radiological diagnosis	Primary	93 (80.1%)	1 (3.0%)	0.453
	Metastasis	1 (6.0%)	15 (45.5%)	
	Benign	7 (12.9)	0	
	Unsure	15 (0.86%)	17 (51.5%)	
Number of renal tumor	Solitary	116	27	<0.001
	Multiple	0	6	
Enhancement pattern	Homogeneous	25	10	0.206
	Heterogeneous	91	23	
Location	Unilateral	116	27	<0.001
	Bilateral	0	6	
Growth pattern	Exophytic	75	5	<0.001
	Endophytic	41	28	
Component	Solid	112	33	0.363
	Cystic	4	0	

### Treatment and Pathology of SPRCC

For the 113 patients with localized RCC, local treatment modalities utilized for renal cancers were partial nephrectomy in 62 (54.9%) patients, radical nephrectomy in 48 (42.5%) patients, and ablation in one (0.88%) patient, and no local treatment was administered in four (3.5%) patients. The reasons why patients do not perform surgical treatment of RCC included advance in stage (four patients, 80%) and postoperative complications (one patient, 20%) of extrarenal malignancies. Among the 73 patients with synchronous SPRCC, 15 patients (20.5%) underwent surgery for multiple organs simultaneously, and 42 patients (57.5%) were given priority in the treatment of nonrenal tumors. For the five patients with metastatic SPRCC, two patients underwent cytoreductive nephrectomy.

The postoperative pathology of SPRCC patients included clear cell RCC (n = 98, 89.1%), papillary RCC (n = 4), chromophobe RCC (n = 5), Xp11.2 translocation RCC (n = 1), sarcomatoid variants of RCC (n = 1), and unclassified RCC (n = 1).

### Outcome of SPRCC

**Figure 1:**
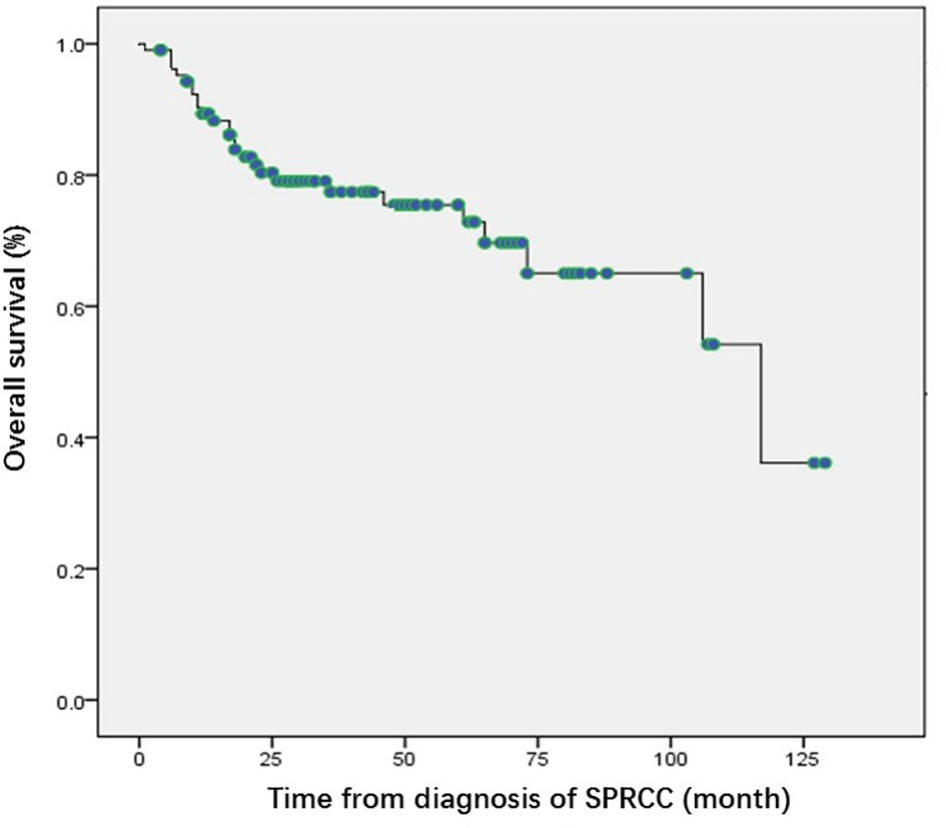
Kaplan Meier curves of overall survival of the patients with second primary renal cell carcinoma. SPRCC, second primary renal cell carcinoma; OS, overall survival.

For SPRCC patients, 106 patients were followed up for a median of 30.5 months (1–129 months). Metastasis of RCC occurred in eight patients; The median time of metastasis was 25.3 months (range, 4–54 months). And, the diagnosis of metastasis was confirmed by puncture biopsy in six patients and by clinical features in the other two patients. At the end of follow-up, a total of 27 deaths had occurred. Nine (8.5%) patients died of kidney cancer and 18 (17%) died of nonrenal malignancies or other causes. The median OS was 117 months (95% confidence interval (CI): 67.4–166.6) for SPRCC patients (**Figure 1**).

Univariate survival analysis showed that for SPRCC patients, female sex, asymptomatic status, no distant metastasis, and surgical treatment predicted a longer OS (**Table 3**). Multivariable analysis showed that OS improved in women with SPRCC (**Table 3**).

**Table 3:** Univariate survival analysis and multivariable analysis of patients with second primary renal cell carcinoma.

Overall survival		Univariate analysis	Multivariable analysis		
		P value	HR	95% CI	P value
Gender	Male	0.003	4.947	1.333,18.363	0.017
	Female				
Smoking history	Yes	0.196	0.717	0.237,2.172	0.557
	No				
Family history of tumor	Yes	0.946	0.587	0.228,1.509	0.269
	No				
Primary nonrenal malignancy	Lung	0.931	1.152	0.395,3.357	0.795
	Others				
Metastasis of other site	Yes	<0.001	1.081	0.133,8.794	0.942
	No				
Symptoms	Yes	<0.001	4.555	0.706,26.396	0.111
	No				
Duration between twice cancer onsets	Synchronous	0.371	0.774	0.299,2.002	0.597
	Metachronous				
Surgery	Yes	<0.001	0.366	0.080,1.662	0.193
	No				

### Differential Diagnosis From Renal Metastases

In addition to SPRCC, RM are another common kidney tumors in cancer survivors. The body mass index (BMI) of SPRCC patients was significantly higher than that of RM patients (23.9 vs. 21.9 kg/m2, p<0.002). In terms of the extrarenal malignancy, a higher percentage of patients with stage I–II was found in the SPRCC group, and a higher percentage of patients with stage III/IV was found in the RM group (p = 0.012). And more patients with RM chose radiotherapy or chemotherapy than those with SPRCC for the treatment of first primary malignancies (73.5% vs. 44.9%, p = 0.01). In addition, more patients with RM had symptoms than patients with SPRCC (p < 0.001) (**Table 1**).

Radiological characteristics of patients with SPRCC and RM are shown in **Table 2**. The imaging features of enhanced CT showed that more RM patients had bilateral, multiple, and endogenic renal masses (p < 0.001) than SPRCC patients, but the solid/cystic features and enhancement patterns were not significantly different between the two groups. However, it was quite difficult to distinguish primary and metastasis on CT scan (P=0.453).

Seven SPRCC patients (5.9%) underwent renal puncture biopsy, with a diagnostic accuracy of 100%, and there were no post-puncture complications. Renal biopsy was performed in 16 RM patients (45.7%), and 15 patients (93.8%) were diagnosed with RM and one patient (6.2%) was pathologically negative (**Table 1**).

## Discussion

RCC has been frequently described as a second cancer event following the diagnosis of other primary cancers (12). Studies based on autopsy have identified a 30 to 42% incidence of other primary malignancies in RCC patients (7). And some cohort studies showed the incidence of other primary malignancies in patients with RCC was about 12-25% (13). Our results demonstrated that the percentage of SPRCC in all RCC was 11.4%. Therefore, SPRCC is not uncommon in clinic.

In terms of the extrarenal malignancies in patients with SPRCC, some studies shared the similar results. Beisland et al. reported that the most common extrarenal malignancies include prostate, bladder, lung, breast, and colorectal cancers, malignant melanoma (MM), and non-Hodgkin’s lymphoma (NHL) (5). Liu et al. reported that increase in the risk of RCC was observed with colorectal, lung, breast, prostate, bladder, nervous system, thyroid gland, and adrenal gland cancers, as well as with MM and NHL (8). And, our study showed that the common types of extrarenal malignant tumors consisted of lung, colorectal, breast, gastric, and gynecological cancers. Some cancers may share similar carcinogenic exposure and genetic predisposition, which can help to screen for the development of a second cancer. Furthermore, heterogeneity in the risk of SPC was substantial across cancer types, which revealed the need to consider the pairs of first and second cancers rather than the overall risk (2). Detection of the relationship between the first primary cancer and SPRCC can reveal novel possible carcinogenic pathways. Therefore, we can take the study of SPRCC as a starting point to explore the new pathogenesis of RCC.

An increased risk of kidney cancer is observed within the first 5 years after the diagnosis of many common cancers (12). Beisland et al. found that the incidence of synchronous SPRCC was 34.6% and that of metachronous SPRCC was 65.4% (5). It is worth noting that synchronous SPRCC accounted for 61.9% of the total patients included in our study, which was much higher than that in the previous study. This may be because our center adopted a more comprehensive systemic examination before surgery, which may have resulted in the detection of cancer in other organs.

When a renal mass is detected in a patients with other cancers before or at the same time, both SPRCC and RM should be suspected. As the kidney is a relatively rare site of secondary malignancy, the data of distinction between SPRCC and RM is usually insufficient (14–16). The differential diagnosis between SPRCC and RM is mainly based on imaging examination. Radiologically, RM is generally small, multiple, bilateral, and wedge-shaped, with less exogenous growth and is generally located in the renal capsule, whereas SPRCC tends to be single, unilateral, and non-wedge-shaped, with an exogenous growth pattern that easily invades the renal capsule (17). Patel et al. compared the CT features of 21 RM patients and 15 SPRCC patients and found that RM was more solid and endogenous than SPRCC (15). Choyke et al. also observed a lack of enhancement in RM (18). Similarly, our study found that bilateral, multiple, and endogenous tumor was observed in more RM patients than SPRCC patients. However, occasionally the differential diagnosis between SPRCC and RM is quite challenging radiologically. Honda et al. reported that the diagnostic accuracy of CT for renal metastatic tumors was 75%, whereas that for primary renal tumors was 93.2% (17). Zhou et al. reported that the accuracy of enhanced CT for the diagnosis of RM was 47.5% (19). Our study showed that the accuracy of enhanced CT in the diagnosis of SPRCC was 80.1% and that of RM was only 45.5%, which is consistent with previous studies. The reasons for the low accuracy of imaging diagnosis may include the following aspects. Some pathological types of RCC, such as papillary RCC and collected duct carcinoma, also have indeterminate enhancement and are poorly defined, which are quite similar with RM on CT (20, 21). In addition, it is worth noting that we found two cases of RM coexisting with primary RCC, which is also known as collision tumor. Because of the complexity of the components of collision tumors, there are great differences in imaging findings, which makes imaging judgment more difficult.

Renal biopsy is an important method to distinguish SPRCC and RM, and it has a high diagnostic accuracy and a low rate of clinically relevant complications (22). Sánchez-Ortiz et al. reviewed the medical records of 100 patients with nonrenal malignancies diagnosed with renal tumors at presentation or follow-up and found that in 74 patients who underwent a biopsy of the renal mass, metastasis was diagnosed in 18 patients, primary renal neoplasms were diagnosed in 41 patients, and 15 patients had a nondiagnostic biopsy (20.3%) (16). In this study, the proportion of renal biopsy in RM patients was 45.7% with 93.8% accuracy and the puncture proportion of SPRCC patients was 5.9% with 100% accuracy. Despite the high diagnostic accuracy, metastases to the kidney were extremely rare in patients presenting with renal masses and another clinically localized malignancy; thus, renal mass biopsies were not indicated for these patients (16). Therefore, renal biopsy is recommended for patients with progressive extrarenal malignancies or suspected RM.

Surgery is the main treatment method for localized RCC (23). However, when determining the treatment for renal tumors in SPRCC patients, the condition of extrarenal malignancies such as T-stage, grade, nodal disease, and previous treatment needs to be considered. The European Association of Urology Guidelines on Renal Cell Carcinoma indicate that elderly and comorbid patients with incidentally detected small renal masses can be managed by active surveillance (AS) (23). However, considering that sporadic RCC has a high risk of metastasis, AS of sporadic RCC should be adopted in patients who cannot tolerate surgery (24). A population-based study using the Surveillance, Epidemiology, and End Results (SEER)-9 database showed that local treatment modalities utilized with SPRCC were partial nephrectomy in 16% of patients, radical nephrectomy in 33% of patients, and ablation in 5.5% of patients, with no local treatment in 18% of patients (12). From our results, the proportion of patients with localized SPRCC who received surgical treatment was 95.6%, which was higher than that in previous studies. This might be related to the high proportion of localized RCC and early stage of extrarenal malignancies in SPRCC patients. In terms of metastatic RCC, the Carmena study shows that the efficacy of targeted therapy is not inferior to that of targeted therapy plus nephrectomy, and it makes urologists tend to be more cautious in their choice of cytoreductive nephrectomy (25). For metastatic SPRCC, the choice of cytoreductive nephrectomy is more contradictory. In our study, among the five patients with metastatic SPRCC, two patients underwent cytoreductive nephrectomy. Due to the small sample, the significance of cytoreductive nephrectomy cannot be confirmed. Further studies were needed to explore the value of cytoreductive nephrectomy in patients with metastatic SPRCC.

Previous studies have reported a poorer prognosis in SPRCC patients than in RCC patients (5, 13). The prognostic factors of simple RCC include stage, histopathological factors (such as nuclear grade, pathological type, sarcomatoid differentiation), and molecular markers (23). However, the prognostic factors of SPRCC are more complex and are also related to the factors of extrarenal malignancies. The results of previous studies on multiple primary malignancies (MPM) show that the interval between the two primary cancers has a positive relationship with prognosis and that patients with metastasis have a reduced survival time (5, 26). The prognosis of MPM is also related to the biological characteristics of each cancer, with a poor prognosis for liver, lung, and pancreatic cancers, and a relatively better prognosis for penile and breast cancers (27). Unlike previous reports for MPM, in our study, univariate analysis indicated that there was no significant correlation between OS and the interval between the two primary cancers, and this may be associated with the high proportion of synchronous SPRCC in this study. Univariate analysis also showed no relationship between OS and the types of extrarenal malignancies because, in this study, extrarenal malignancies and RCC were mainly localized in the early stage, and the follow-up time was limited, which may have affected the analysis of prognosis. RCC staging is also a prognostic factor, which is consistent with previous studies (13). In addition, this study also showed that female, asymptomatic, and surgical patients had a longer OS than the others.

The present study has several limitations. First, the sample size of patients was relatively small, especially for RM patients. Second, this study is retrospective in nature, and hence, recall errors could easily occur and affect the results. Third, the follow-up time for SPRCC patients was relatively short, which affected the analysis of survival-related data.

## Conclusions

In conclusion, SPRCC are not uncommon, and it should be considered during the follow-up of patients with nonrenal malignancy. Surgery is the most important treatment method for SPRCC patients, but it is also necessary to consider the stage, treatment, and prognosis of the extrarenal cancers. It is necessary to distinguish SPRCC from RM. There are some differences between the two groups in terms of clinical features and imaging manifestations, and renal biopsy can be used for diagnosis.

## Data Availability Statement

The raw data supporting the conclusions of this article will be made available by the authors, without undue reservation.

## Ethics Statement

The studies involving human participants were reviewed and approved by the Medical Ethics Committee of Zhejiang Cancer Hospital. The patients/participants provided their written informed consent to participate in this study.

## Author Contributions

SZ was responsible for the concept and design of the study. JC, NQ, HW, ZW, and YH dealt with the clinical data. JC performed the statistical work and drafted the manuscript. YH provided the figures and tables. All authors revised the manuscript.

## Conflict of Interest

The authors declare that the research was conducted in the absence of any commercial or financial relationships that could be construed as a potential conflict of interest.

## Publisher’s Note

All claims expressed in this article are solely those of the authors and do not necessarily represent those of their affiliated organizations, or those of the publisher, the editors and the reviewers. Any product that may be evaluated in this article, or claim that may be made by its manufacturer, is not guaranteed or endorsed by the publisher.
